# Restoration and Efficiency of the Neural Processing of Continuous Speech Are Promoted by Prior Knowledge

**DOI:** 10.3389/fnsys.2018.00056

**Published:** 2018-10-31

**Authors:** Francisco Cervantes Constantino, Jonathan Z. Simon

**Affiliations:** ^1^Program in Neuroscience and Cognitive Science, University of Maryland, College Park, College Park, MD, United States; ^2^Department of Electrical and Computer Engineering, University of Maryland, College Park, College Park, MD, United States; ^3^Department of Biology, University of Maryland, College Park, College Park, MD, United States; ^4^Institute for Systems Research, University of Maryland, College Park, College Park, MD, United States

**Keywords:** speech processing, auditory cortex, magnetoencephalography, stimulus reconstruction, speech envelope

## Abstract

Sufficiently noisy listening conditions can completely mask the acoustic signal of significant parts of a sentence, and yet listeners may still report the perception of hearing the masked speech. This occurs even when the speech signal is removed entirely, if the gap is filled with stationary noise, a phenomenon known as perceptual restoration. At the neural level, however, it is unclear the extent to which the neural representation of missing extended speech sequences is similar to the dynamic neural representation of ordinary continuous speech. Using auditory magnetoencephalography (MEG), we show that stimulus reconstruction, a technique developed for use with neural representations of ordinary speech, works also for the missing speech segments replaced by noise, even when spanning several phonemes and words. The reconstruction fidelity of the missing speech, up to 25% of what would be attained if present, depends however on listeners’ familiarity with the missing segment. This same familiarity also speeds up the most prominent stage of the cortical processing of ordinary speech by approximately 5 ms. Both effects disappear when listeners have no or little prior experience with the speech segment. The results are consistent with adaptive expectation mechanisms that consolidate detailed representations about speech sounds as identifiable factors assisting automatic restoration over ecologically relevant timescales.

## Introduction

The ability to correctly interpret speech despite disruptions masking a conversation is a hallmark of communication (Cherry, 1953). In many cases, contextual knowledge poses an informational advantage for a listener, so as to successfully disengage the masker and restore the intended template signal (Shahin et al., 2009; Riecke et al., 2012; van Wassenhove and Schroeder, 2012; Leonard et al., 2016; Cervantes Constantino and Simon, 2017). Usually, relevant information is available from multimodal sources and/or low-level auditory and higher level linguistic analyses, although it remains unclear how and which factors are most effective in assisting speech restoration under natural conditions. Recently, cortical network activity profiles consistent with phonemic restoration, the effect where absent phonemes in a signal may nonetheless be heard (Samuel, 1981, 1996), have been identified in binary semantic decision tasks (Leonard et al., 2016), yet factors that bias into one or the other of two perceptual alternatives remain unclear. At the algorithmic level, there is evidence that such restorative processes may be influenced by contributions from audiovisual integration cues (Crosse et al., 2016), lexical priming (Sohoglu et al., 2012), and within the auditory domain, by predictive template matching (SanMiguel et al., 2013). At the computational level, proposals include the deployment of intentional expectations about temporal patterns in sound (Nozaradan et al., 2011; Tal et al., 2017), and the use of mental imagery as a weak form of perception (Pearson et al., 2015).

In order to affect ongoing speech percepts, outcomes from such mechanisms would have to be readily accessible before and during missing auditory input. These type of contributions potentially entail (i) generation of a provisional template of the forthcoming speech, (ii) that the template be stored in a compatible format with the internal representation of ongoing sound, and (iii) that they are later subject to point-wise matching – in what has been termed the *zip metaphor* (Grimm and Schröger, 2007; Tavano et al., 2012; Bendixen et al., 2014). In addition, the contribution by such putative mechanisms in enhancing the neural representation of speech may allow a speed up of cortical processing during integration (van Wassenhove et al., 2005).

Here, we test how a string of natural speech tokens spanning several words may be represented cortically, even if entirely removed and replaced by stationary masking noise – under different levels of informational gain provided by prior knowledge of the masked elements. Prior research has shown that information from missing consonants can be inferred from cortical activity sustained over brief (∼100 ms) noise probes, by their similarity to responses to the original consonant sounds, such as a single fricative (Leonard et al., 2016). We use the fact that the low-frequency envelope of speech spanning several words indexes the acoustic signal’s slow changes over time and is known to phase-lock neural activity in auditory cortex, as measured by magnetoencephalography (MEG) and electroencephalography (EEG) (Giraud et al., 2000; Ding and Simon, 2012b; Zion Golumbic et al., 2013; Di Liberto et al., 2015). Because of its timescale, the low-frequency envelope of speech typically reveals attributes such as the patterns of syllabic lengths and loudness changes, as well as prosodic information including intonation, rhythm, and stress cues. We hypothesize that by repeating the strings of speech tokens, and controlling for the extent of repetition, one can manipulate listeners’ cortical ability to develop detailed predictions about forthcoming elements in these long sentences. More repetitions would allow the generation of more detailed templates for those tokens, to serve for a point-wise matching when later, spontaneous maskers disrupt the same string of tokens. If neurally instantiated, then the process may be investigated by testing how well the missing speech token can be decoded from the cortical signals representing it, despite the lack of related acoustic input. Furthermore, because the template would be formed prior, one may also investigate the possibility that cortical representations of highly repeated speech stimuli are facilitated by accelerated processing times.

To address these hypotheses, we employ a pair of complementary systems-based neural analysis methods. In one case, we analyze neural responses to reconstruct the stimulus speech envelope (Mesgarani, 2014), an approach that has been successfully applied in auditory electrophysiology (Mesgarani et al., 2009; Ramirez et al., 2011), EEG/MEG (Ding and Simon, 2012a; O’Sullivan et al., 2015), electrocorticography (Pasley et al., 2012; Leonard et al., 2016), and fMRI (Naselaris et al., 2011). In the case of speech restoration, electrophysiological responses have been used to re-create the acoustic representation of the stimulus using a data-driven decoder that effectively recovers the spectrogram of a missing consonant (i.e., substituted by noise) from listeners’ cortical activity (Leonard et al., 2016). Complementary to this reconstruction analysis, temporal response function (TRF) analysis uses an acoustic representation of the stimulus to predict neural responses. This forward model permits direct analysis of cortical latencies involved in natural speech processing, the most prominent of which occurs between 100 and 180 ms, consistent with the latency of the evoked response M100 component (Cervantes Constantino et al., 2017). We investigated the possibility of related adaptations, such as reduced cortical latencies, under the same prior knowledge conditions employed in the decoding analyses, since faster processing has been observed in situations where additional context facilitates perceptual integration of incoming speech (van Wassenhove et al., 2005; van Wassenhove and Schroeder, 2012). In addition, similar task-related cortical plasticity changes in stimulus-response mappings are often observed at the neuronal level (Fritz et al., 2003; David et al., 2012) and would represent a potential biophysical basis for restorative mechanisms given the present task demands. The forward model applied to the MEG was then used to address whether and how similar adaptations might be reflected at the whole brain level.

We provide evidence that the speech temporal envelope is better reconstructed when listeners have obtained more knowledge about a particular speech sequence, and, critically, that this effect applies even in the case where the speech itself is absent, having been replaced entirely with noise. The data also show that cortical latencies in the processing of clean speech can be reduced by several milliseconds when the listener has obtained detailed knowledge about that particular speech sequence. Overall, the results suggest improved efficiency in accessing dynamic neural representations of low-level features of frequently experienced speech, indicated by both faster stimulus encoding and endogenous restorative processes that reflect a neural representation of the missing speech.

## Materials and Methods

### Participants

Thirty-five experimental subjects (19 women, 21.3 ± 2.9 years of age [mean ± SD]), with no history of neurological disorder or metal implants, participated in the study. Data from one additional subject was not included, due to excessive artifacts caused by a poor fit with the MEG helmet. Each subject received monetary compensation proportional to the study duration (approximately 1.5 h). This study was carried out in accordance with the recommendations of the UMCP Institutional Review Board with written informed consent from all subjects. All subjects gave written informed consent in accordance with the Declaration of Helsinki. The protocol was approved by the UMCP Institutional Review Board.

### Stimuli and Experimental Design

Sound stimuli were prepared with the MATLAB^®^ software package (MathWorks, Natick, MA, United States) at a sampling rate of 22.05 kHz, and consisted of a recorded poem (“A Visit from St. Nicholas,” Moore or Livingston, 1823) obtained from an online archive^1^. In addition to the narrated contents, the spoken verses transmit intonation, rhythm, and stress cues, all amenable for encoding as prosodic information units, and all predisposed to potential cortical restoration extending over multiple syllables. Each of the 14 verses (each verse being a quatrain of four lines) in the poem were separated and considered as individual stimuli. In order to probe the contribution of prior experience to cortical coding, the four stimulus blocks presented to each subject, each containing 64 stimuli (i.e., 256 lines), had some of stimuli repeated multiple times, as follows. For the first block, a verse/stimulus from the first half of the poem was chosen to be a “High” frequency stimulus, with sufficient repetitions to make up half of the block’s stimulus presentations (32/64). Similarly, other verses were chosen as “Medium” and “Low” frequency stimuli, which were repeated for a quarter (16/64) and an eighth (8/64) of the block’s stimulus presentations, respectively. The remainder of the block was filled with “Control” stimuli, namely the four remaining verses presented either one, two, or four times within the block. This category represents, for missing speech, the case for which the listener would have insufficient prior experience with the specific speech segment to promote restoration; for non-missing speech, forward model analysis of this case acts as a control for comparing latencies of more frequently presented stimuli.

Silent intervals (gaps) in the narration were reduced to approximately equalize stimuli durations (range: 13.1–13.6 s). Stimuli were randomized in order and concatenated in time. For the second block, the same procedure was followed using material from the second half of the poem. Blocks 3 and 4 consisted of the same stimuli used as in 1 and 2, respectively, but with a different randomized order and different placement of noise probes (see below). The procedure was recreated with different randomizations for each subject, resulting in a total of 35 different stimulus sets of about 1 h each in total duration. Importantly though, the usage of particular stimuli at a given repetition level was controlled across participants, resulting in seven groups of five listeners each that underwent the same “High,” “Medium,” “Low,” and “Control” stimuli selection.

For each stimuli, two to four spectrally matched noise probes of 800 ms duration each were applied at pseudo-random times with a minimum 2.5 s between probe onsets. Noise onset times were selected from a pool of values indicating articulation onset times (e.g., syllables), obtained as the envelope rising slope maxima. Thus, 768 noise probe samples were presented per experiment, and each was individually constructed by randomizing phase values across the specific frequency-domain phase information contained in the underlying speech stimulus that would have occurred at the same time as the masker noise, yielding a noise with equal spectral amplitude characteristics (Prichard and Theiler, 1994). The original speech content occurring during the same time was removed entirely and substituted with this spectrally matched noise, at a power signal level matching that of the excised clean original. Subjects listened to the speech sounds while watching a silent film. To ensure attention to the auditory stimulus, after each probe, they were instructed to report via a button press whether they understood what the speaker meant to say during the noise. The button presses are not analyzed here.

### Data Recording

We recorded neural responses using MEG, a non-invasive neuroimaging technique well-suited to measure dynamical neural activity from human cortex, and especially from auditory cortical areas. Such recordings typically demonstrate time-locked neural responses to speech low frequency modulations, especially of the acoustic energy envelope, with remarkable temporal fidelity (Ding and Simon, 2012b). MEG data were collected with a 160-channel system (Kanazawa Technology Institute, Kanazawa, Japan) inside a magnetically shielded room (Vacuumschmelze GmbH & Co. KG, Hanau, Germany). Sensors (15.5 mm diameter) were uniformly distributed inside a liquid He dewar, spaced ∼25 mm apart. Sensors were configured as first-order axial gradiometers with 50 mm separation and sensitivity >5 fT.Hz^-1/2^ in the white noise region (>1 kHz). Three of the 160 sensors were magnetometers employed as environment reference channels. A 1-Hz high-pass filter, 200-Hz low-pass filter, and 60-Hz notch filter were applied before sampling at 1 kHz. Participants lay supine inside the magnetically shielded room under soft lighting, and were asked to minimize movement, particularly of the head.

### Data Processing

#### Pre-processing and Sensor Rejection

The time series of raw recordings from the MEG sensor array were be submitted to a fast implementation of independent component analysis (Hyvärinen, 1999), from which two independent components were selected for their maximal proportion of broadband (0–500 Hz) power (because of the ∼1/*f* power spectrum of typical neural MEG signals, these components are dominated by non-neural artifacts). These independent components, combined with the physical reference channels, were treated as environmental noise sources arising from unwanted electrical signals not related to brain activity of interest, and were removed using time-shifted principal component analysis (TS-PCA) (de Cheveigné and Simon, 2007). Sensor-specific sources of signals unrelated to brain activity were reduced by sensor noise suppression (SNS) (de Cheveigné and Simon, 2008b).

### Data Analysis

To analyze low-frequency cortical activity, recordings were band-pass filtered between 1 and 8 Hz with an order-2 Butterworth filter, with correction for the group delay. A blind source separation technique, denoising source separation (DSS) (de Cheveigné and Simon, 2008a), was used to construct components (virtual channels constructed of linear combinations of the sensor channels), ranked in order of their trial-to-trial reproducibility, trained *only from clean speech presentations*, and used as described below.

#### Stimulus Reconstruction

The ability to reconstruct the speech stimulus envelope from recorded neural responses was used to measure the dynamical cortical representation of perceived speech. Decoders were separately estimated based on either reproducible or “reference” activity as ranked by DSS, with two such pairs of decoders: the first pair trained on responses to speech and the second pair trained on responses to noise, as described in the following. The first decoder of each pair was based upon the first three DSS components (i.e., with highest reproducibility across instances of listening to *clean speech*). These highly reproducible components were used to train an optimal linear decoder designed to reconstruct the envelope of the stimulus that was presented under normal speech listening conditions but absent (though perhaps expected) under noise listening conditions. That the reproducible neural activity is generated by auditory cortex is reflected in a DSS component topography consistent with auditory responses arising from temporal cortices (Supplementary Figure S1). The last three DSS components (with the lowest reproducibility from the same clean speech dataset), were similarly used to train the second linear decoder for each pair, used as a reference, i.e., to estimate baseline performance. Each decoding procedure produced a corresponding reconstructed stimulus time series whose similarity with the corresponding speech envelope was assessed via Pearson’s *r* correlation coefficient. Each similarity score was, respectively, designated as reproducible (*r*_e_) and reference (*r*_f_). This referencing procedure is necessary to obtain a baseline in decoding performance since time series’ lengths varied across conditions (as a result of the different repetition rates and verses involved); otherwise, there would be positive biases in *r* for shorter sequences, irrespective of underlying relationship to the stimulus. The appropriate pairs of decoders were applied separately to their respective neural responses, to clean speech and to noise. Noise edge and button-press-related segments were excluded from all analysis. To the extent that a noise-only response can be used to reconstruct an absent but expected stimulus (better than baseline performance) reflects the presence of neural activity consistent with a representation of the acoustically absent speech.

To compute reconstruction effect sizes, each of the Pearson’s *r* pairs (reproducible versus reference activity) were transformed to Cohen’s effect size *q* (Cohen, 1988) by the transform $q=\frac{1}{2}\left(\ln\frac{1+r_{e}}{1-r_{e}}-\ln\frac{1+r_{f}}{1-r_{f}}\right)$ for both kinds of responses. Relative effect sizes (speech versus noise reconstruction) were computed by the fraction *q*_2_/*q*_1_ of reconstruction effect sizes given the stimulus presentation conditions above (expressed as percentages), where *q*_1_ denotes the effect size obtained from reconstructions of clean speech from neural activity following clean speech, and *q*_2_ the effect size from reconstructions of clean speech from neural activity arising from the noise probe (devoid of speech). Absolute effect sizes during noise presentations were used for statistical analysis.

#### Temporal Response Function of Stimulus Representation

The input–output relation between a representation *S*(*t*) of auditory stimulus input and the evoked cortical response *r*′(*t*) is modeled by a TRF. This linear model is formulated as:

$${r^{\prime }}_{\mathrm{pred}}(t)=\sum_{\tau }TRF(\tau )S(t-\tau )+\varepsilon (t)$$

where ε(*t*) is the residual contribution to the evoked response not explained by the linear model. As stimulus representation, the envelope was extracted by taking the instantaneous amplitude of each channel’s analytic representation via the Hilbert transform (Bendat and Piersol, 2010), with sampling rates reduced to 1 kHz, transformed to dB scale. The response was chosen to be either the first or second DSS component (fixed for each subject; Supplementary Figure S1), according to which one produced a TRF with a more prominent M100_TRF_, a strong negative peak with ∼100 ms latency (Ding and Simon, 2012a).

#### Statistical Analyses

For reconstructions, one-way repeated measures ANOVA were run across the four levels: “Control,” and “Low,” “Medium,” and “High” repetitions, in order to examine differences between their related means overall. Cortical latency of the TRF was determined by the M100_TRF_ latency. Peak delays with respect to control conditions were determined by cross-correlations of the TRF in the “Control” versus all other repetition conditions. The resulting peak delays were then submitted to a non-parametric one-tailed two-sample Kolmogorov–Smirnov test for differences in the underlying delay populations.

## Results

### Neural Transformations Facilitated by Prior Experience Result in Improved Ability to Reconstruct Missing Speech From Noise

**FIGURE 1:**
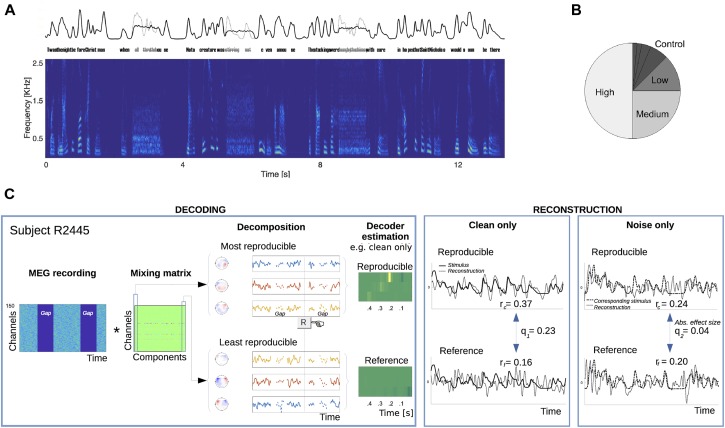
Present/absent speech stimulus protocol and cortical speech envelope reconstruction algorithm procedure. **(A)** Speech material from a single poem was continuously presented to 35 listeners over the course of an hour, except that every 4–5 s, some speech was replaced with spectrally matched noise (0.8 s duration; three replacements shown). This manipulation removes all critical temporal modulation for that duration. Top: example of the slow envelope of continuous speech with occasional missing speech replaced by stationary noise in black; the envelope of the removed missing speech is in gray. Bottom: spectrogram of the continuous speech with missing speech replaced by stationary noise. **(B)** For any participant, a verse selection was chosen to be repeated more than any other (50% of all verse presentations) and denoted as having a “high” repetition rate. Another verse selection was similarly chosen to be repeated with a “medium” repetition rate (25% of all verse presentations). Additional verses were repeated less frequently: 12.5% (“low”), 6.25%, etc. Verses presented 6.25% or less often were grouped together as “Control.” The frequency designation of verses was counterbalanced across subjects. **(C)** The stimulus protocol served to allow analysis of reconstruction performance for both types of response, to clean speech and to stationary noise without speech. In the *decoding* step, a subject’s multichannel response was decomposed into components, ordered by trial-to-trial reproducibility during speech listening (see Section “Materials and Methods”). The top three waveform components (with corresponding topographies shown) served to train a *reproducible* neural activity-based decoder to estimate the clean speech low-frequency envelope. Correspondingly, the bottom three components trained a *reference* (baseline) decoder. Separate pairs were trained according to whether the speech was present (as shown in this panel) or absent and replaced by noise (not shown). In the *reconstruction* step, the appropriate pair of reproducible and reference decoders were applied to the neural responses to speech or noise, respectively. Results of the envelope reconstruction algorithm are shown for a representative subject.

Connected syllables/words within a narrated poem were replaced by noise bursts of fixed duration. Each static noise probe was constructed with the same spectrum as the replaced speech segment (Figure 1A), and therefore lacked critical temporal modulations, e.g., in the low-frequency (2–8 Hz) envelope. Low-frequency fluctuations present in natural, unmasked speech typically generate time-locked auditory cortical activity recorded by MEG (Giraud et al., 2000; Ding and Simon, 2012b). Multichannel recordings thus contain information about dynamic neural representations of speech, and so may in turn serve as the basis to train decoding models that reconstruct dynamic features of the presented speech signal, e.g., its envelope. Linear decoders mapping from MEG responses to the stimulus speech envelope were estimated per subject, and their envelope reconstruction performance (in estimating the original speech signal) quantified. Crucially, decoders were either trained from recordings of clean speech presentation intervals, or, separately trained from intervals when only static noise was delivered. In all cases, performance was tested with respect to the corresponding clean speech signal at that part of the poem, whether actually delivered or not. To test whether acoustic presence is a necessary condition for reconstruction of continuous speech, listeners were exposed to extensive repetitions of some verses (each verse being a quatrain of four lines), and less frequent repetitions (or none at all) to the rest (Figure 1B). To limit confounding effects from specific properties of particular verses, counterbalanced subgroups heard different sets of verses for each repetition frequency, i.e., the five participants in each subgroup experienced the same verses with identical repetition frequencies, but the next subgroup heard a different set of verses for each repetition frequency.

The hypothesis was tested using an index of speech reconstruction, Cohen’s *q*, estimated using a two-step process (Figure 1C). For each subject, first, a data-driven response mixing matrix was obtained from responses to clean speech only, a procedure that serves to decompose multichannel timeseries into their most and least reproducible components relevant to natural speech processing. These most and least reproducible sets served to generate reconstruction models of the original speech signal, estimating optimal and baseline, respectively, reconstruction performance levels in the subsequent stage. Second, one pair of reproducible and reference decoders was trained from recording intervals where speech was delivered, and separately, another pair was similarly trained from intervals presenting only noise. In all cases, the reconstruction models targeted the envelope of the normal speech stimulus, even when that speech was absent (but perhaps expected) under the noise listening conditions. In the final step of the reconstruction stage, the *q* index was computed from comparing the appropriate pair of reproducible versus reference decoding performances. A separate *q* index was computed for responses to speech and to noise, and for each repetition frequency condition.

**FIGURE 2:**
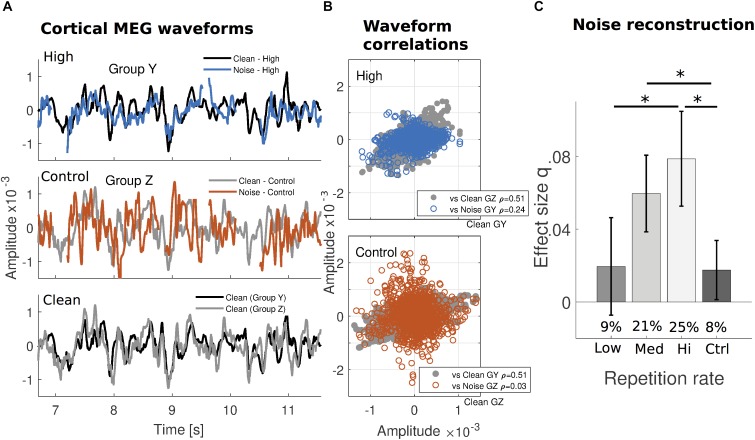
Increased robustness of cortical reconstructability to missing speech under substantial prior exposure. **(A)** The median cortical MEG response waveforms from one cross-validation subject subgroup (group Y; *N*_Y_ = 5) exposed to a specific verse with High frequency, are contrasted with those from a different subgroup (group Z; *N*_Z_ = 5) exposed to the same verse but infrequently (Control). Within the High repetition rate subgroup (top), signals generated by clean speech (black) and static noise replacing the missing speech (blue) appear to covary more than for the Control repetition rate subgroup (middle: gray and red, respectively). The waveforms in response to clean speech from both subgroups are replotted together for comparison (bottom). (Gaps in the responses to noise are due to intervals when the speech was never replaced by noise, or during noise onset or button presses.) **(B)** Correlation coefficients indicate that, while clean speech responses are similar to each other for both subgroups (gray, same data in both plots), for noise intervals, they are much more similar to the clean responses only when the verse is frequently repeated (blue, top), but not when infrequently repeated (red, bottom). For a comparison from other subgroups using High and Low repetition rate conditions, see Supplementary Figure S2. Median waveforms use the dominant auditory response component (see Supplementary Figure S1) and are determined for each time point over the subject group. **(C)** The missing dynamic speech envelope may be reconstructed from responses to noise, with performance of about 25% of that obtained under clean conditions (percentages inset for each bar). As indicated by the absolute *q* index dependence on presentation frequency using the noise-trained decoders, this result is not a consequence of any clean-trained decoders dependence on frequency. Error bars indicate confidence intervals for the means (Bonferroni-corrected α level).

Differences in the time-locked auditory cortical responses to clean speech and to noise intervals, by repetition frequency of the verse they belonged to, are illustrated in Figure 2A (for two different subject subgroups that experienced the same verses at two repetition frequencies). Over the span of several seconds, the neural waveforms in response to (aggregated) noise intervals appear more comparable to those in response to clean speech when the verse in question was frequently repeated in a subgroup (High; upper graph) than not (Control; middle graph). This is demonstrated by the timeseries’ correlation coefficients between waveform pairs across the entire verse (Figure 2B). Because the repetition frequency of particular verses is counterbalanced across subjects, the analogous comparison for High versus Low repetition conditions (Supplementary Figure S2) uses different subgroups.

In terms of reconstruction performance, sentences that were maximally repeated (High repetition rate) over the hour-long session resulted in greatest relative performance in reconstruction of the envelope of the missing speech: approximately 25% of the performance for actual speech presented without any masking. Less exposure resulted in further reductions in relative performance (Medium: 21%, Low: 9%, and Control: 8%, respectively), down to the floor level in the case of masked speech with which the listener had little or no prior experience (Figure 2C; percentages inset). Because this measure is relative to clean speech reconstruction, a measure of reconstruction from noise alone was tested separately, using Cohen’s *q* to quantify the effect size. Effect sizes in reconstruction of the missing speech envelope were confirmed to display a similar pattern as with relative performance (High: 0.079 ± 0.013; Medium: 0.060 ± 0.011; Low: 0.020 ± 0.013; Control: 0.018 ± 0.008) (Figure 2C). A one-way repeated measures ANOVA with four repetition levels was performed to determine whether decoding success of the linear model of the envelope significantly changed across conditions. *q* index results for reconstructions exclusively using noise intervals showed that the sphericity condition was not violated [Mauchly test, x^2^(5) = 6.322; *p* = 0.276]. The subsequent ANOVA resulted in a significant main effect of repetition frequency [*F*(3,102) = 8.070; *p* = 7.1 × 10^-5^]. *Post hoc* pairwise comparisons using Bonferroni correction revealed that this increased exposure to speech significantly improved the stimulus reconstruction effect size from Low and Control repetition rate conditions to High (*p* = 2.5 × 10^-3^ and *p* = 7.7 × 10^-4^, respectively), and also from Control to Medium (*p* = 7.7 × 10^-3^).

### Expedited Auditory Cortical Processing of Natural Speech

**FIGURE 3:**
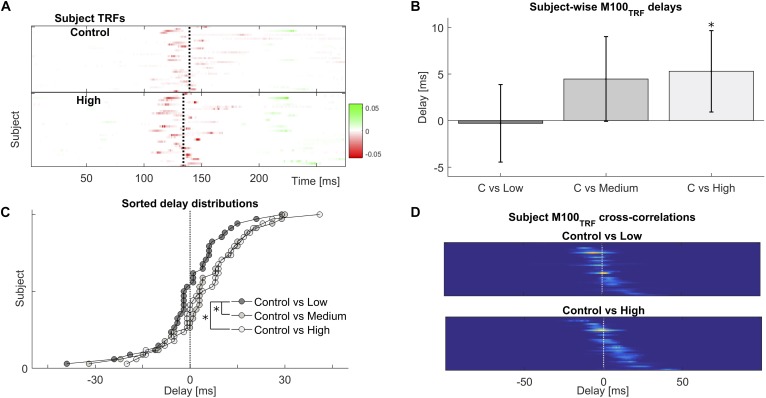
Frequent repetitions of natural speech speed-up their cortical processing. **(A)** Temporal response functions across participants reveal a common cortical processing step, referred to as the M100_TRF_, typically occurring about 100 ms after a speech envelope fluctuation (red colored features near the vertical dotted lines). **(B)** Depending on familiarity with the speech tokens, the same processing step may shift in time: processing of frequently repeated speech occurs about 5 ms earlier than for novel or sparsely presented sentences, within subjects. **(C)** Across subjects, the distribution of relative delays is consistently biased towards positive (earlier) values for the most extreme repetition conditions. **(D)** Illustration of how shifts within subjects were obtained, by cross-correlating individual M100_TRF_ peak profiles obtained per condition in each subject.

The TRF is a linear model used to predict the dynamics of the neural response to sound input, given a representation of the stimulus such as the acoustic envelope. Its characteristic peaks, and especially their polarity and latencies, are indicative of the progression of neural processing stages akin to the distinct generators of evoked responses to simple sounds such as pure tones (Ding and Simon, 2012a,b; Cervantes Constantino et al., 2017), but with the advantage of being directly derived from the neural processing of continuous natural speech. We examined the effect of prior exposure on the TRF’s temporal structure in general, and also for the most prominent peak, the M100_TRF_, occurring 100–180 ms post envelope change (Figure 3A). When a given speech sequence was listened to repeatedly, a significant within-participant latency shift of 5.3 ± 2.2 ms earlier was observed for $M100_{\mathrm{TRF}}^{\mathrm{High}}$ versus $M100_{\mathrm{TRF}}^{\mathrm{Control}}$ peaks [*t*(33) = 2.387; *p* = 0.023], indicating expedited cortical processing cortical for more familiar stimuli (Figure 3B). Across participants, the differences between repeated (High, Medium, and Low) and baseline (Control) levels, in terms of maxima in their cross-correlation functions, were shown to arise from significantly different distributions (*D* = 0.294; *p* = 0.043) (Figures 3C,D), suggesting that prior experience by repeated presentations effectively speeds up cortical processing even as early as 100 ms latency.

## Discussion

We present evidence of dynamic envelope coding of missing natural speech, by means of stimulus reconstruction methods applied to auditory cortical responses. This occurs as long as when there has been a history of repeated, frequent exposure to the original missing speech, suggesting that prior experience facilitates access and maintenance of a detailed temporal representation of the stimulus even though absent as low-level input. In addition, we find that cortical processing dynamics timescales are reduced by about 5 ms under similar prior experience conditions, for natural speech processing.

Spectrogram reconstructability of noise-replaced phonemes (e.g., fricatives) has been demonstrated when subjects interpret the remainder of the single word accordingly (Leonard et al., 2016). Such endogenous activity may arise from top-down modulations of auditory cortical areas (Petkov et al., 2007; Petkov and Sutter, 2011) with the effect of modulating perceptual processing, including, the ability to entrain to speech signals (Ding et al., 2013), to optimize detection performance (Henry and Obleser, 2012), and to support auditory illusions (Riecke et al., 2009). Under the umbrella of *attractive temporal context effects* (Snyder et al., 2015), a group of facilitatory mechanisms including perceptual hysteresis and stabilization (cf. Kleinschmidt et al., 2002; Pearson and Brascamp, 2008; Schwiedrzik et al., 2014), auditory restoration effects improve perceptual invariance in the face of discontinuously fluctuating, broadly cluttered environments. The involvement of storage-based reactivation in perceptual processes, including attention, is an area of active research (Backer and Alain, 2012, 2014; Zimmermann et al., 2016). We therefore provide evidence for reactivation mechanisms based on prior learning and storage of speech information, at the level of its temporal structure.

### Access and Format of Stored Auditory Representations: Hierarchical Models

Encoding of speech and other stimuli into sensory memory, the function of primary sensory areas that integrates analysis and storage of stimulus features by relevance (Cowan, 1984; Weinberger, 2004), has been argued to assist in the ability to restore missing fragments of a sound source, e.g., as an internal replay of the fragment during phonemic restoration (Shinn-Cunningham, 2008). Examples of implicit auditory memory in sensory and perceptual encoding (Snyder and Gregg, 2011) are observed in repetition-based improved detection of arbitrary noise constructs, and on neural covariates of this improvement (Agus et al., 2010; Andrillon et al., 2015).

Foreknowledge of acoustic features allows adaptation to a likely communication source, as shown by, e.g., facilitation with advance notice regarding the identity of a forthcoming instrument (Crowder, 1989), and by preferential activation in auditory association areas specific to speaker familiarity (Birkett et al., 2007). Such differential activation, given variable rates of sensory update, suggests that prior experience history of a dynamic sound pattern may influence its later representation: with few initial updates, storage at short intervals is associated with posterior superior temporal cortex, but over time, activation may take place at inferior frontal cortex (Buchsbaum et al., 2011). This progression is consistent, in memory terms, with readout from sensory buffers taking place at high temporal resolution under low-level representation formats; coarser temporal resolutions are instead attained at stores that operate under categorical, higher order feature codes (cf. Durlach and Braida, 1969; Winkler and Cowan, 2005). For perception, progression hierarchies are core features of models such as reverse hierarchy theory, which proposes that fast perception (e.g., when understanding speech in noise) is by default based on high-level cortical representations (Ahissar et al., 2009), except for specific conditions as systematic stimulus repeats, where information about fine temporal detail may then also be utilized (Nahum et al., 2008). Hierarchical models can be useful inasmuch they identify stages by which feed-forward general stimulus template extraction steps are completed, and they specify roles for feedback activity from higher areas (Kumar et al., 2007). In hearing their application includes, e.g., pitch and spectral envelope analyses, where top-down information serves to adapt effective processing time constants over lower areas that encode more temporally refined information (Kumar et al., 2007; Balaguer-Ballester et al., 2009).

Thus, a general prospect of these models is to determine the extent to which the natural hierarchy in sensory input might map to the anatomical hierarchy of the brain. In temporal terms, another application refers to an interesting distinction between “percept” versus “concept” representations of an environmental variable, namely, transient versus enduring representations (Kiebel et al., 2008). Because only the enduring (e.g., >1 s) representations have the capacity to shape how lower level representations may evolve, in the language of dynamical systems, they are seen as control parameters: consolidation of a “concept” automatically constrains where the trajectories of representations at subordinate processing levels may unfold autonomously (Kiebel et al., 2008). The role of prior knowledge in the cortical hierarchy of speech perception and representation, in particular with regard to the acoustic envelope, is a matter of current research interest (Sohoglu and Davis, 2016; Di Liberto et al., 2018). Hierarchical approaches therefore appear as a suitable framework to bridge findings of low-level endogenous representations with a mechanistic account of phonemic and speech restoration.

### Phonological Structure and the Role of Auditory Retrieval Processes in Noise Listening

A tenet of speech restoration phenomena posits the use of prior abstractions or “schemata” that remain represented online, e.g., when an expected stimulus fails to occur, and are better resolved with increased familiarity (Hubbard, 2010). From the operational perspective, these are based upon the phonological structure of natural speech: representations first involve recognition of phonological structure (what is being heard), and second, that words be stored into verbal working memory using phonological code stores (Wagner and Torgesen, 1987). For efficiency reasons, the codes for lexicon storage and for the later retrieval probing process itself may be both the same, with phonologically similar words grouped together in the lexicon in “neighborhoods,” as indicated by “phonological awareness” models from the developmental literature (e.g., Nittrouer, 2002; Lewis et al., 2010). In our results, this framework would indicate a hypothetical process for words replaced by noise probes where (1) each high-frequency word has been established as a competitive representative of its respective neighborhood store, (2) retrieval is however constrained to operate endogenously, primarily from available contextual information, and (3) the tempo of retrieval would be coordinated at a timescale superordinate to that of phonological units, e.g., by prosodic and sentential information. In this scenario, the temporal envelope of missing speech, learned by prior experience, may coordinate the probe-triggering process.

Besides phonological structure, identification of auditory persistence processes unprompted by sensory input (Intons-Peterson, 2014) may involve self-directed imagery tasks (Bailes, 2007; Meyer et al., 2007), where activation levels in the planum temporale correlate with self-reported levels of imagery engagement and perceived vividness (Zatorre et al., 2009); both auditory imagery and rehearsal may be subserved by auditory association cortex areas in general (Meyer et al., 2007; Hubbard, 2010; Martin et al., 2014). Furthermore, in a bimodal stimulation study, transient activity from superior temporal cortex was shown to be critical at the beginning of the auditory retrieval process, but sustained planum temporale activity was involved overall (Buchsbaum et al., 2005). This is consistent with the interpretation that in such retrieval processes, task-relevant stimulus features may be maintained at (re)activated domains within the sensory representational space (Kaiser, 2015). The notion that both representation and maintenance involve overlapping processes (Hubbard, 2010) is supported by findings of reactivation, at retrieval, of sensory regions active during perception (Wheeler et al., 2000), and with auditory verbal imagery (McGuire et al., 1996; Shergill et al., 2001). The emerging, increasingly multimodal field of neural representations sustained during mental imagery may further support crucial clinical applications (Pearson et al., 2015).

### Adaptive Dynamics of Speech Encoding and Representation During Masking

As related to speech, two main mechanisms indicate a correspondence of results with generative neural models (Pouget et al., 2013). First, the finding that cortical processing is sped up, under the same circumstances that promote restoration of speech-related neural activity, suggests that active, task-related endogenous processes directly optimize low-level speech processing with experience. A plausible mechanism for this is in promoting increased excitability of higher level neural populations. Second, our results indirectly support the suggestion that auditory “image” formation may entail activity consistent with that elicited by the original sound input (Janata, 2001; Martin et al., 2017), and whose temporal precision, and other related feature properties, may vary depending on factors such as context and experience (Janata and Paroo, 2006). The effect of frequent “refreshing” seen here may relate to the auditory memory reactivation hypothesis (Winkler and Cowan, 2005), where individual sound features can be effectively stored, along with neighboring sound patterns and sequences, when represented altogether by the auditory system as regularities. Over the course of presentation, high-level verse regularities may be continually learned, represented, and accessed, serving as referents. The current findings suggest that masker noise occurrences may be translated into missing values in the same low-level feature format as the low frequency envelope. While this does not preclude other dynamic features of speech to contribute to reactivation processes, such as higher order linguistic elements (e.g., Näätänen and Winkler, 1999; van Wassenhove and Schroeder, 2012; Di Liberto et al., 2015; Kayser et al., 2015), the key neural property of natural sound encoding via temporally based acoustic representations is underscored by its active maintenance during noise gaps as a function of prior experience.

## Author Contributions

FCC conceived, designed, and performed the experiments, analyzed the data, and prepared the manuscript figures. JZS supervised the research. Both authors wrote the manuscript text.

## Conflict of Interest Statement

The authors declare that the research was conducted in the absence of any commercial or financial relationships that could be construed as a potential conflict of interest.

## Footnotes

1 https://archive.org/details/AVisitFromSt.Nicholas-ByClementClarkeMoore-NarratedByGrantRaymond
