# Supplementary material for: Restoration and Efficiency of the Neural Processing of Continuous Speech Are Promoted by Prior Knowledge
Source: Front Syst Neurosci. 2018 Oct 31;12:56. doi: 10.3389/fnsys.2018.00056 (PMC6220042; doi:10.3389/fnsys.2018.00056)
Supplement: Supplementary file 1 [file Data_Sheet_1.PDF]

Supplementary Material

Restoration and efficiency of the neural processing of continuous speech are promoted by prior knowledge

Francisco Cervantes Constantino, Jonathan Z. Simon*

*** Correspondence:** Jonathan Z. Simon: jzsimon@umd.edu

Supplementary Figure 1. Field topographies associated with the dominant response component for every subject (*N*=34), where component order within a subject was determined by the order of reproducibility of neural activity (across instances of listening to clean speech). These topographies predominantly represent neural activity originating in bilateral auditory cortex. Such dominant response components were always among the top two reproducible components, and chosen for the strength of the temporal response function (TRF) profile (see Methods) at 100-180 ms latency. Topography field strength is in standardized (z-score) units.

Supplementary Figure 2. (**A**) Median cortical MEG waveforms from a subject subgroup (N_W=5) exposed to a different verse (than in Fig. 2) with High frequency, contrasted with those from a fourth subgroup (N_X=5) less frequently exposed to the same verse (Low frequency). Results are consistent with those shown in Figure 2: within the High repetition rate subgroup (top), signals generated by clean speech (black) and static noise replacing the missing speech (blue) appear to covary more than for the Low repetition rate subgroup (middle, grey and red respectively). The waveforms in response to clean speech from both subgroups are replotted together for comparison (bottom). (**B**) Correlation coefficients indicate that, while clean speech responses are similar to each other for both subgroups (grey, same data in both plots), for noise epochs they are much more similar to the clean responses only when the verse is frequently repeated (blue, top), but not when infrequently repeated (red, bottom).
