# Supplementary figures and images for: Restoration and Efficiency of the Neural Processing of Continuous Speech Are Promoted by Prior Knowledge
Source: Front Syst Neurosci. 2018 Oct 31;12:56. doi: 10.3389/fnsys.2018.00056 (PMC6220042; doi:10.3389/fnsys.2018.00056)

**A****Cortical MEG waveforms**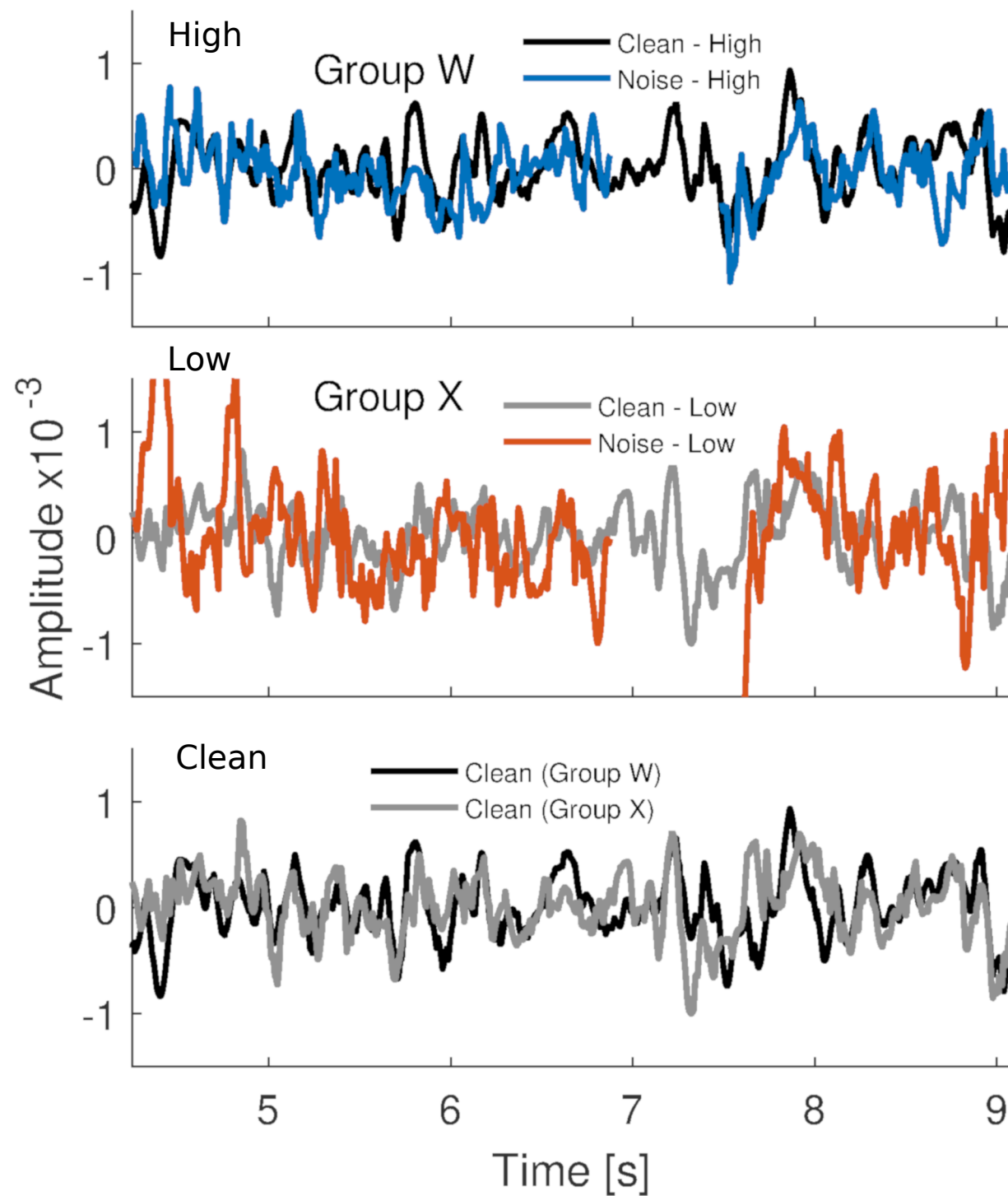**B****Waveform correlations**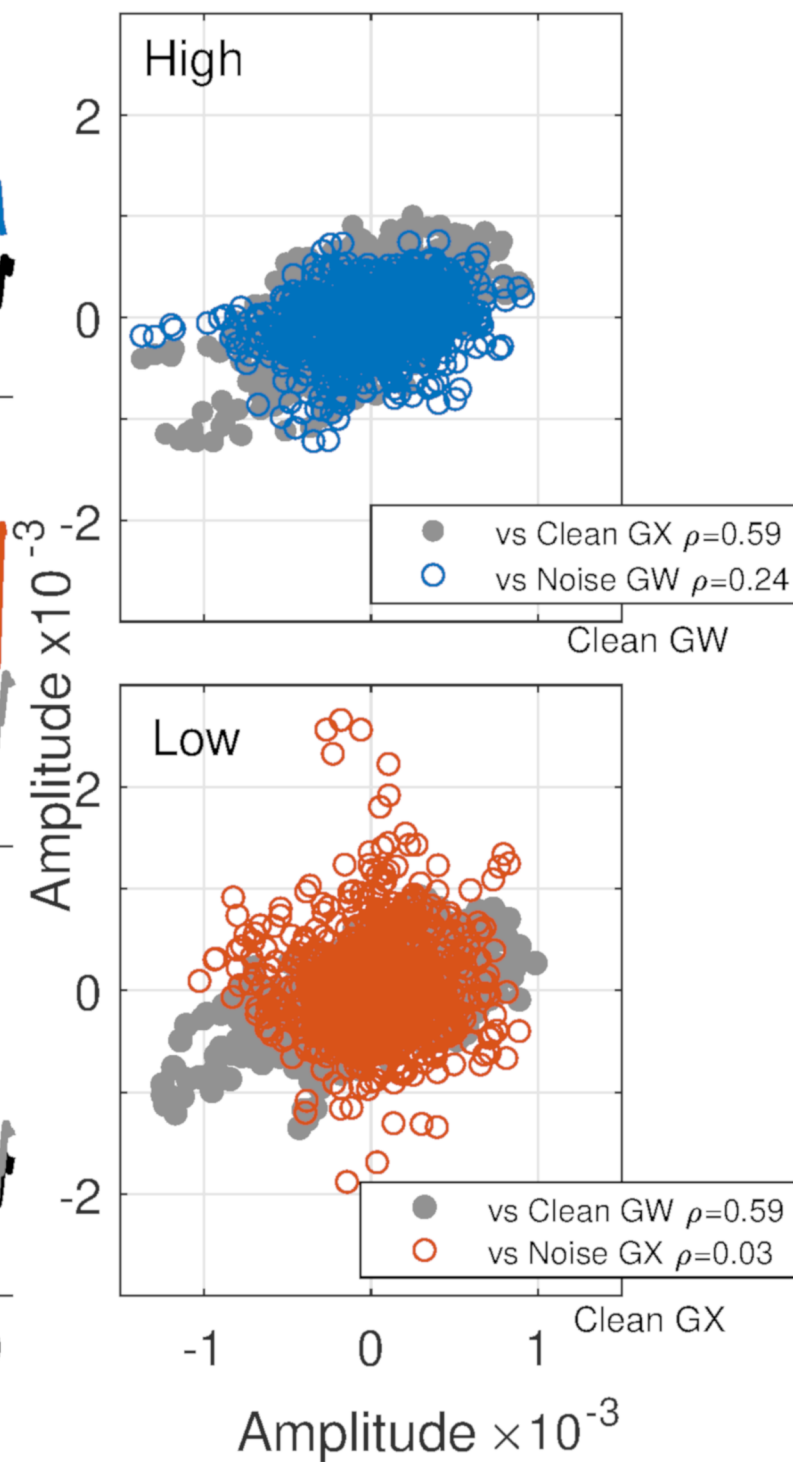

Supplement: Supplementary file 3 [file Image_2.PDF]
