# Supplementary material for: Restoration and Efficiency of the Neural Processing of Continuous Speech Are Promoted by Prior Knowledge
Source: Front Syst Neurosci. 2018 Oct 31;12:56. doi: 10.3389/fnsys.2018.00056 (PMC6220042; doi:10.3389/fnsys.2018.00056)
Supplement: Supplementary file 2 [file Image_1.PDF]

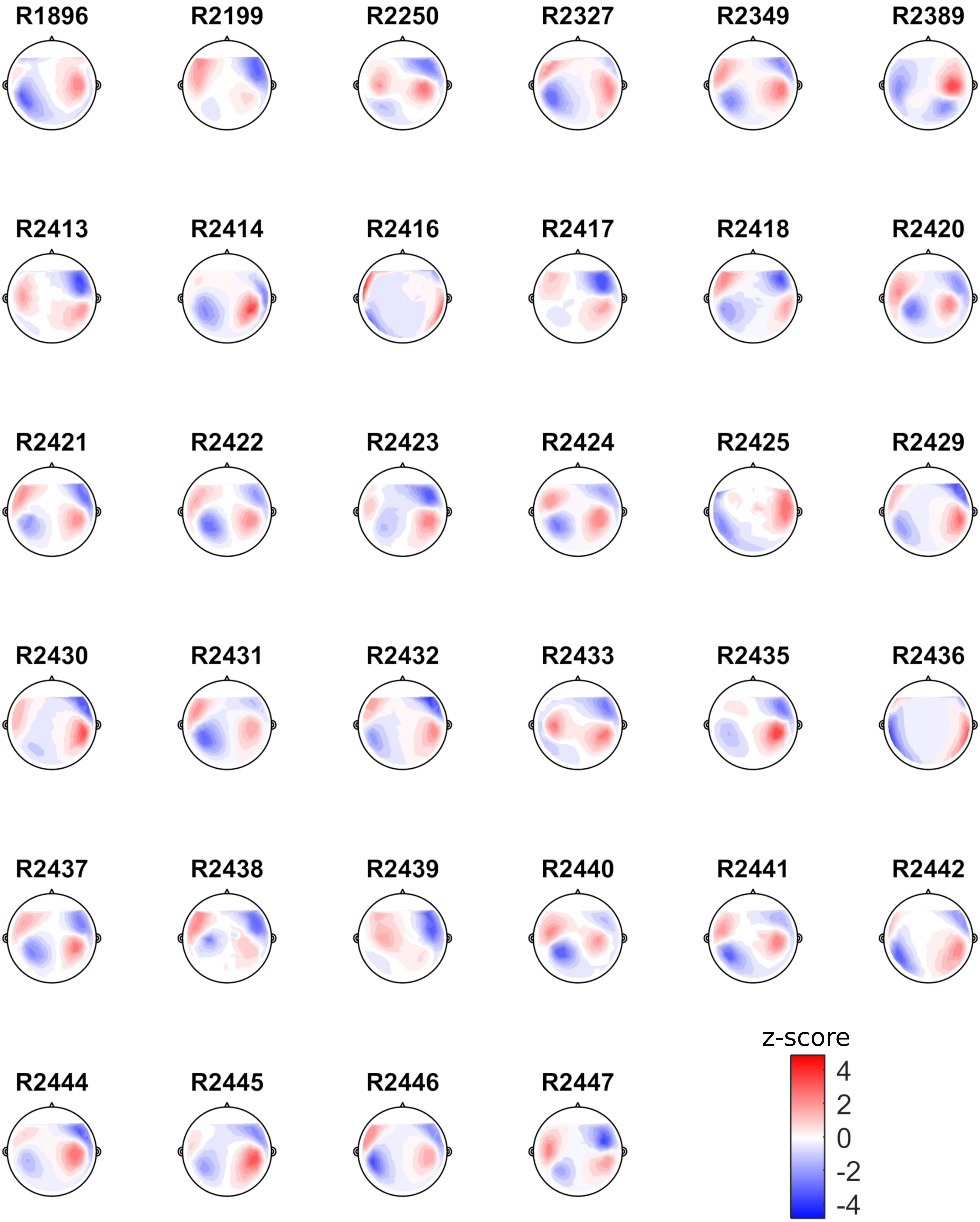
R1896
R2199
R2250
R2327
R2349
R2389
R2413
R2414
R2416
R2417
R2418
R2420
R2421
R2422
R2423
R2424
R2425
R2429
R2430
R2431
R2432
R2433
R2435
R2436
R2437
R2438
R2439
R2440
R2441
R2442
R2444
R2445
R2446
R2447
z-score
4
2
0
-2
-4
